# Injuries of the Head—Bloodletting in Extravasation

**Published:** 1879-04-20

**Authors:** E. F. Wells

**Affiliations:** Minster, O.


### *INJURIES OF THE HEAD—BLOODLETTING IN EXTRAVASATION.*

BY E. F. WELLS, M.D., MINSTER, O.

When symptoms denoting intra-cranial extravasation arise, the clot of blood external to the vessels is already an accomplished fact. The advocates of bloodletting tell us that the abstraction of ten or twenty ounces of blood from a vein of the arm or leg, or from the jugular, or from an artery, will prevent its increase, promote the absorption of the clot, and grant immunity from inflammation; and the theory is seductive; besides having the support of the writings and practice of several generations of surgeons, and we all know how irresistible is the rule of brilliant and exalted example in matters surgical.

Let us for a moment consider the physical condition of the parts involved. If it be the middle meningeal artery or one of its branches, or one of the larger arteries at the base of the brain that is lacerated, it will be found that rarely or never is the artery completely severed; but on the contrary the artery will usually be found punctured or incised by some sharp fragment of the vitreous table or the instrument inflicting the original injury. The only exception to this rule is found when the middle meningeal artery runs completely or nearly so in a bony canal, or the artery involved is the subject of atheromatous or calcareous degeneration.

If the hemorrhage be from the numerous bloodvessels of the pia mater, the conditions are somewhat changed. Here the division may be complete or partial. Within the brain, the opening is generally of limited extent and with ragged edges, but seldom will it be found that the vessel has been completely divided. Capillary hemorrhage usually occurs from vessels completely divided.

Hemorrhage may cease from :

1st. When the vessel is completeiy divided.

*a.* The contraction of the vessel.

*b.* The retraction of the inner coat.

*c.* The formation of a clot.

*d.* The pressure of the clot lying external to the artery.

*e.* The pressure of foreign bodies.

*f.* The failure of the *vis a tergo.*

*g.* The intervention of art.

2d. When the vessel is partially divided.

*a.* The contraction of the vessel.

*b.* The formation of a clot.

*c.* The pressure of the clot lying external to the artery.

*d.* The pressure of foreign bodies.

*e.* The failure of the *vis a tergo.*

*d.* The intervention of art.

It needs no extended reasoning to show that, of all the ways in which the flow of blood is stopped, only contraction of the vessel and the failure of the *vis a tergo* can possibly be influenced by bloodletting, and, as I propose showing further on, this influence has been, in times past, greatly over-estimated or probably does not exist.

That the muscular wall of the blood vessels is paralyzed by the shock to the nervous mass, caused by the extravasation, is proven by the dilated vessels and congestion found to exist. This condition is remedied in *every case* by the spontaneous efforts of nature ; and in obedience to a general physiological law, viz : *Every relaxation of living muscular tissue is followed by contraction, when the cause of the relaxation is removed.* That bloodletting will diminish the force of the intra-cranial circulation, I readily admit ; yet in judging of the applicability of this remedy, we must keep in view the fact that its infiuence is only potent in case the smaller vessels are implicated, and further that the mischief has already reached its acme, and the opening in the vessel has probably been closed by pressure before the accession of symptoms that lead to the employment of bleeding.

"But," say the phlebotomists, "the good effects of our procedure do not end here. We have, in general bloodletting, a most valuable remedy in the further treatment of the case; for it will not only promote absorption but will also prevent the supervention of inflammation."

It is needless for me to state in this place, that these statements are mere assertions, without any foundation in fact, and incapable of verification. On the other hand we are justified in inferring from analogy, that such is not the case.

It must be granted that there is little perceptible difference between an ecchymosis in the cellular tissue under the skin, and one within the cranium. No one, in the first instance, expects to promote absorption and lessen the risk of having a supervening inflammation in the part, by means of bloodletting. The universal use of mild compression is proof of its usefulness, and it may be that the compression exerted upon intra-cranial clots by the force of the circulation and the effused serum in the neighborhood, aided by the elasticity of the parts, are directly beneficial in promoting absorption.

Keeping in view these facts, I cannot but be of the opinion that bloodletting, if not injurious, is totally ineffectual; and that the practice of Pott, the Coopers, Brodie, and our own Gross should be, in this particular, reinvestigated under the light of modern science and theories.

